# AWARENESS OF HEALTH RISKS OF CAREGIVING AMONG PRIMARY PHYSICIANS AND THE CAREGIVERS THEMSELVES

**DOI:** 10.1093/geroni/igz038.794

**Published:** 2019-11-08

**Authors:** Sara Carmel, Yaacov Bachner, Aya Biderman

**Affiliations:** 1 Ben Gurion University of the Negev, Bee Sheva, Israel; 2 Faculty of Health Sicences, Ben Gurion University of the Negev, Beer-Sheva, Israel, Israel

## Abstract

Literature indicates that family primary caregivers (FPC) to severely-ill patients are at high risk to incidence of physical and mental morbidity, especially if they are old themselves. The purpose of these studies was to examine the FPC’s awareness to their own health risks, and primary physicians’ awareness to the importance of locating, following and providing preventive treatment to FPC in their clinic. Participants included 202 FPC older spouses with average age 70.7 (SD=8.33), and 68% women, and primary physicians (N=201) with average age of 48.5 (SD=11.22) and 53.5% women. Among the FPC, awareness to the health risks of caregiving was higher the greater was the caregiving burden, the worse was their self-rated health, the severer was the patient’s disease, as well as the lower was the number of children and among women and spouses. Awareness to importance of medical surveillance was low. Among physicians, awareness to the risks of caregiving was highest among physicians who received their diploma in Israeli universities, and to specialists in family medicine. Their awareness was higher to the FPC’s susceptibility to mental risks rather than to physical risks. Physician’s awareness to the efficiency of medical surveillance of the FPC was high but their actual performance of it was low. This finding is in accordance with the FPC’s report about a low level of interest in their own health among their primary physicians. The importance of awareness to location, surveillance and preventive treatment of FPC should be promoted in medical education, and primary medical services.

